# Supplementary figures and images for: Caco-2/HT29-MTX co-cultured cells as a model for studying physiological properties and toxin-induced effects on intestinal cells
Source: PLoS One. 2021 Oct 7;16(10):e0257824. doi: 10.1371/journal.pone.0257824 (PMC8496855; doi:10.1371/journal.pone.0257824)

pSGLT1

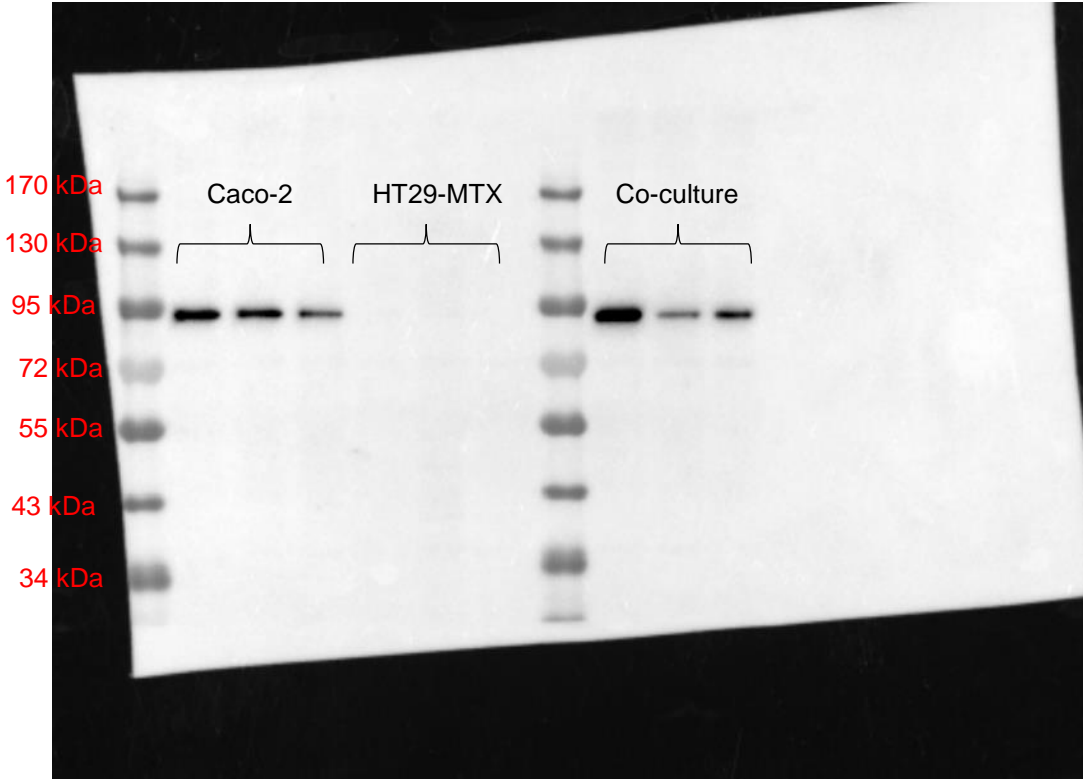

SGLT1

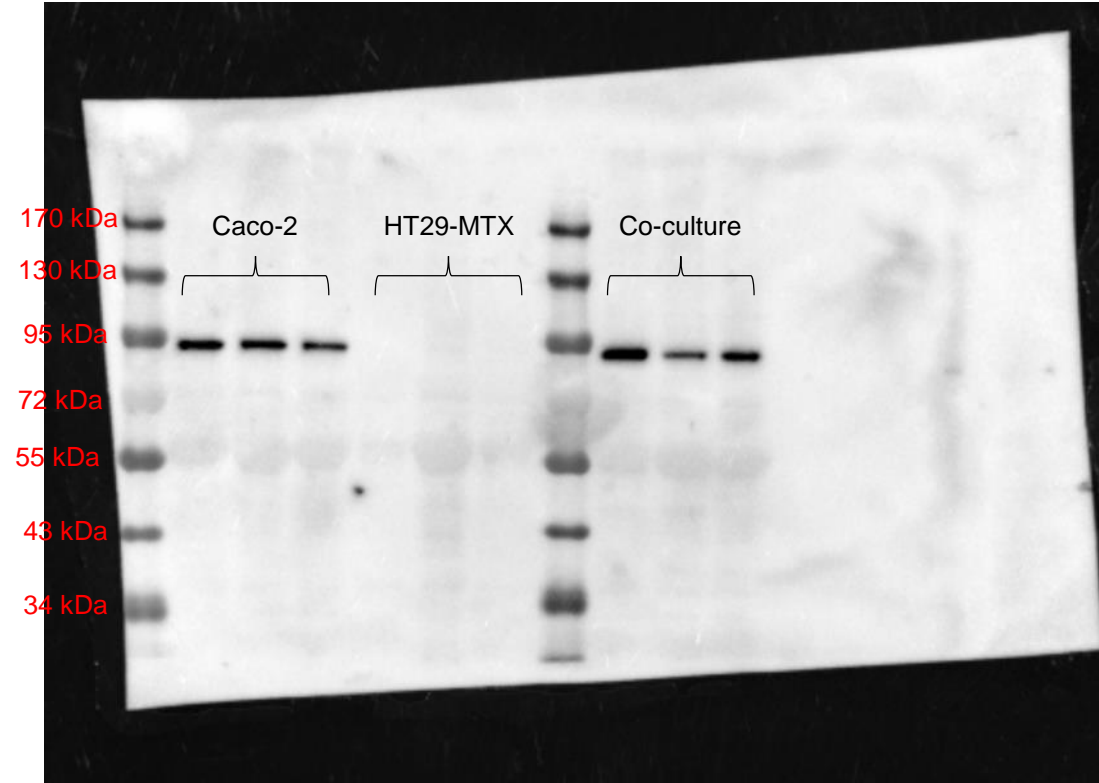

villin

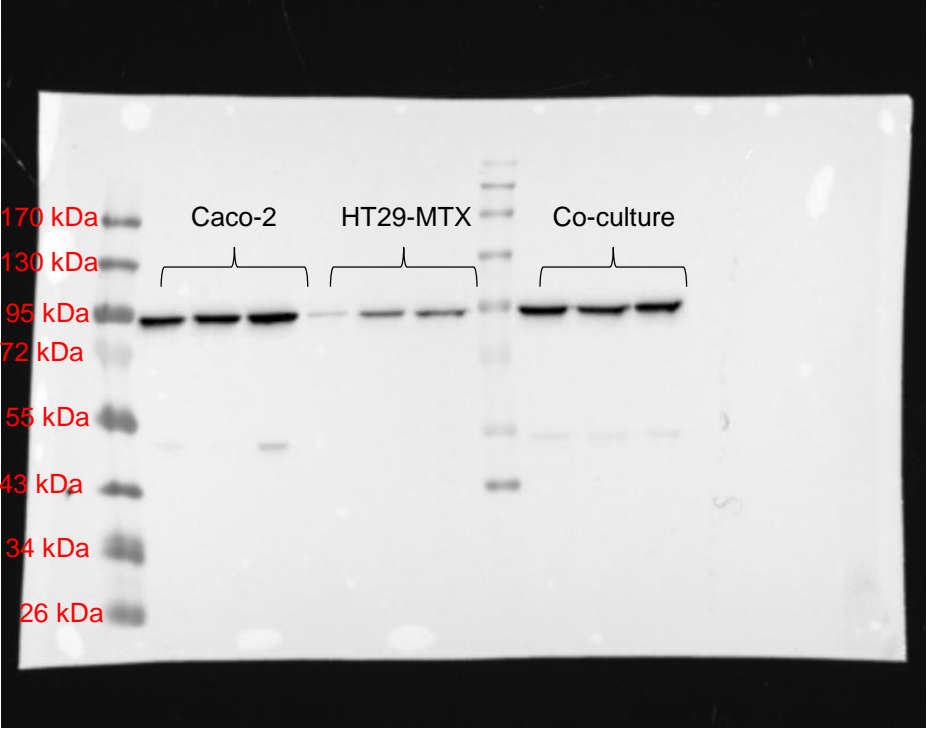

total protein

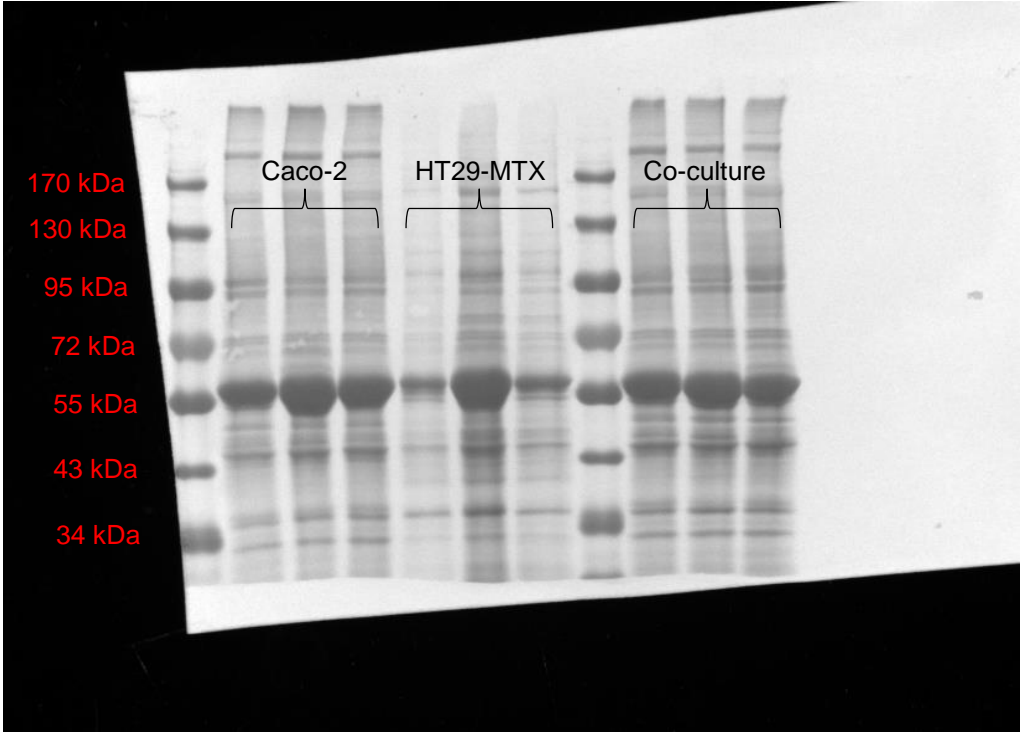

PepT1

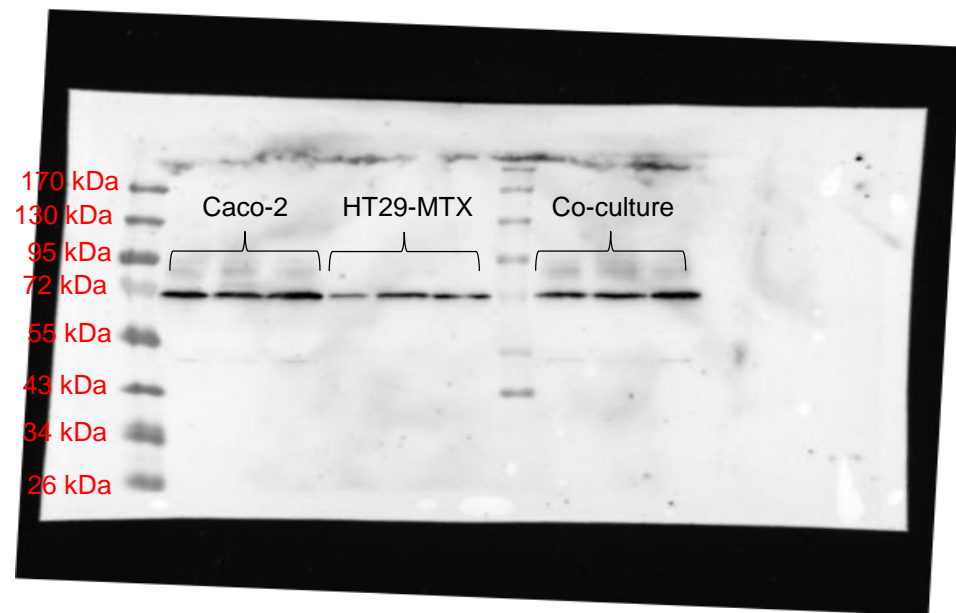

CFTR

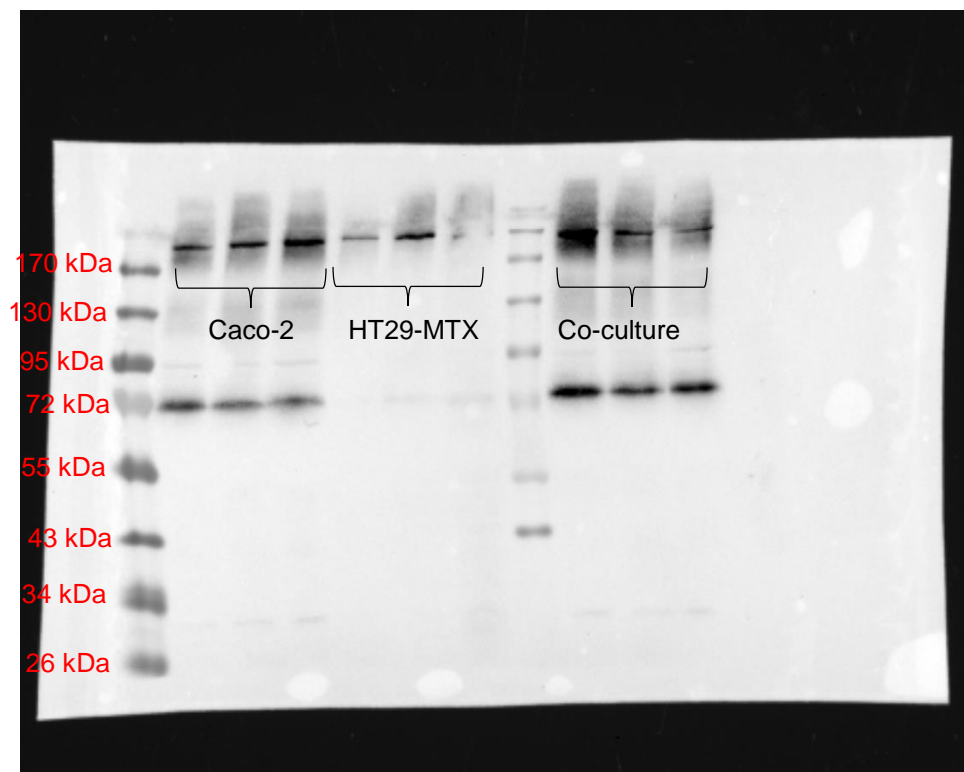

Supplement: S1 Raw images — (PDF) [file pone.0257824.s002.pdf]
